# Micro-, Meso- and Macrofactor Relationships in Nursing Turnover: Insights From Survey and Interview Data

**DOI:** 10.1155/jonm/5078305

**Published:** 2025-07-01

**Authors:** Jennifer Sumner, Hui Wen Lim, Brigitte Woo, Yee Wei Lim, Margaret Lee, Hwee Chyi Yeo, Amartya Mukhopadhyay

**Affiliations:** ^1^Medical Affairs—Research, Innovation & Enterprise, Alexandra Hospital, National University Health System, Singapore; ^2^Department of Healthcare Redesign, Alexandra Research Centre for Healthcare in a Virtual Environment (ARCHIVE), Alexandra Hospital, National University Health System, Singapore; ^3^Alice Lee Centre for Nursing Studies, Yong Loo Lin School of Medicine, National University of Singapore, Singapore; ^4^Yong Loo Lin School of Medicine, National University of Singapore, Singapore; ^5^Department of Nursing, Alexandra Hospital, National University Health System, Singapore; ^6^Department of Medicine, Division of Respiratory and Critical Care Medicine, National University Hospital, Singapore

**Keywords:** job satisfaction, nurse retention, qualitative research, turnover

## Abstract

**Background:** Nurse retention is a persistent and complex problem. Using a system approach, we aimed to understand what is currently impacting nursing turnover and the interplay between the micro-, meso- and macrolevel factors.

**Materials and Methods:** We surveyed and interviewed current and former nurses using a convenience sampling approach. The survey *n* = 479, which targeted working nurses, included questions on job satisfaction and workload. For interviews, we recruited both practising nurses and nurse leavers *n* = 35. The interviews explored individual experiences and perspectives on nursing and what influences nursing turnover. The data were analysed through a system lens, exploring the relationship between an individual's behaviour, interactions and relationships (microlevel), the organisational environment, including policies and regulations (mesolevel) and the social, economic, political and cultural norms within which individuals and organisations reside (macrolevel).

**Results:** Results showed a complex interplay of micro-, meso-, and macrofactors shaping the nursing experience. The survey data revealed poor satisfaction with work-life balance (51%), control over work (43%) and remuneration (43%). Over half (53%) of the participants were considering leaving their organisation, and 36% were contemplating exiting the profession due to exhaustion (74%), inadequate staffing (72%), feeling undervalued (66%), low pay (61%) and excessive pressure (58%). Qualitative interviews revealed negative personal interactions, generational conflicts, unmet or poor expectations of nursing (microlevel), limited autonomy, administrative burdens, poor work-life balance (mesolevel), integration challenges and prohibitive immigration policies for foreign nurses, negative public perceptions and the impact of education on nursing expectations (macrolevel). Furthermore, we found that these factors do not operate in isolation; rather, micro-, meso- and macrolevels interact.

**Conclusions:** Our study underscores the importance of adopting a system approach to understand and address nursing retention issues. Examining micro-, meso- and macrofactors and the interplay between these levels is essential in developing targeted interventions to improve nurse retention.

## 1. Background

Healthcare worker shortages, especially in nursing, remain a significant challenge. By 2030, the healthcare sector could face a shortfall of 18.2 million workers [1]. Contributing factors include a shortage of graduating nurses, coupled with an ageing nursing workforce exiting the profession and ageing populations with higher nursing needs [2]. In some countries retention and recruitment are also complicated by a reliance on foreign labour [3]. For example, competition with other countries for the nursing workforce pool, cultural and language differences that make integration challenging, and the diversion of resources away from domestic workforce training. Consequently, major efforts have gone into understanding nurse attrition and developing retention strategies [4–7].

Workforce retention is crucial to functional and effective health services, as understaffing impacts care quality and patient outcomes [8]. Reasons for leaving the nursing profession are multifaceted and complex. For example, job satisfaction, which encompasses factors such as remuneration, peer relationships and managerial support, is consistently associated with nurses' intentions to stay or exit the profession [4, 6]. Other factors, including stress levels, workload and opportunities for professional growth, are also associated with decisions to leave [4, 6].

Over the years, various interventions have targeted the issue of nursing retention, including incentives for long service, support for education and career development, transition programmes for newly qualified nurses and greater recognition of overseas qualifications [9, 10]. Although targeting individual and organisational level factors has been a key to advancing retention strategies, the macrolevel factors influencing nursing retention are less explored. For example, visa requirements, political situations and national policies impact work conditions and influence workforce retention in other professions [11, 12]. Critically, micro–meso–macro factors do not operate independently. Instead, they interact and influence each other [13]. Therefore, a holistic view is essential for a complete understanding and intervention design in nursing retention.

Given the complex and ongoing challenge of nurse retention, our study aims to expand evidence on nursing turnover by using a system approach [14, 15]. Specifically, we use a system approach to understand what currently impacts nursing turnover and the interplay between the micro-, meso- and macrolevel influences.

## 2. Materials and Methods

We conducted a parallel, convergent, mixed-method study comprising of a survey and interviews with current and former nurses. The survey focused on micro- and mesolevel factors, including job satisfaction and workload. The interviews explored these topics in greater detail and additionally explored macrolevel factors. This study is reported according to the Consensus-Based Checklist for Reporting of Survey Studies (CROSS) and the Consolidated Criteria for Reporting Qualitative Research (COREQ) [16, 17].

### 2.1. Participants

For the survey, we adopted a convenience sampling approach. The study data were collected anonymously. Practicing nurses were recruited through electronic email blasts through the National University Health System (NUHS), Singapore. NUHS comprises of one tertiary, two secondary and seven primary care institutions. We aimed to reach a broad cross section of practicing nurses within the NUHS, Singapore. As the primary aim was to explore general patterns of job satisfaction and work experiences rather than test a specific hypothesis, a formal sample size calculation was not performed. The survey was launched in English on Qualtrics^XM^ (https://qualtrics.com), an online survey platform. Prior to launch, the survey was tested for clarity among the study team. The survey link was emailed on 17th March 2023, and a reminder email blast was sent on 24th March 2023. Participants were also encouraged to share the survey link to other colleagues, and nursing management circulated the link amongst their teams (i.e., snowball recruitment). The survey eventually closed on 5th June 2023. The eligibility criteria for the survey were as follows:

### 2.2. Inclusion Criteria

The inclusion criteria include the following:

• Qualified and currently practicing nursing staff from any setting (i.e., hospital, community, public and private) and across all job grades and
• Adults aged 21 years or older

### 2.3. Exclusion Criteria

The exclusion criteria include the following:

• Participants from unrelated non-nursing fields or
• Nursing students or those no longer practicing nursing or
• Basic care assistants (e.g., nursing assistant) or equivalent

For the interviews, a convenience sampling approach was adopted. First, those completing the survey were asked if they would like to be contacted for an interview and had the option of sharing their contact information. Second, a study advert was circulated amongst known contacts. Third, the human resources department at Alexandra Hospital shared the study information with nurses who had left the institution. Fourth, participants taking part in the interview were also asked if they had colleagues willing to participate (i.e., snowball). Participants were contacted by email or phone messaging. The eligibility criteria were the same as the survey, except we also recruited nurses who had left the profession. We used a mixture of recruitment strategies to capture a range of perspectives, including both currently practising nurses and those who had left the profession. This approach also enhanced the richness and credibility of our qualitative findings by recruiting from diverse sources. We did not perform a formal sample size calculation, as is typical for qualitative research. Instead, we followed the principle of data saturation—the point at which no new themes or insights were emerging from additional interviews—to guide our recruitment. A widely accepted approach in qualitative research methodology was adopted [18].

Nurses who were willing to partake in interviews shared their contact information with the research team through email or phone messaging. A research team member (with no direct relationship with the participant) followed up with the potential participant to discuss the project, take informed consent and arrange a convenient time for the interview.

### 2.4. Survey Outcome Measures

The survey was in English and comprised of five main sections: Demographics—intention to leave, job satisfaction and workload. The content validity of the survey was checked prior to launch through review and discussion on survey items within and outside the project team. The gathered feedback helped to improve the clarity of the survey.

The McCloskey/Mueller Satisfaction Scale (MMSS, 31-items) was used to measure job satisfaction [19]. This validated scale is used extensively in nursing research and healthcare administrative settings to measure job satisfaction. Numerous studies have confirmed its internal consistency (Cronbach's alpha > 0.80) and construct validity [19]. While the MMSS has not been formally revalidated in the Singapore context, it has been used locally among Singapore-based nursing populations, including in studies examining job satisfaction in mental health and acute care settings, supporting its contextual relevance [20–22]. Scale domains include satisfaction with extrinsic rewards, scheduling, family/work balance, coworkers, interaction, professional opportunities, praise/recognition and control/responsibility; each item is rated on five-point Likert scale. We included an additional two questions on intention to leave, assessed using a five-point Likert scale (*strongly agree* to *strongly disagree*): (1) I am thinking about changing organisation but will continue nursing; (2) I am thinking about leaving the nursing profession and a further 10 additional questions on workload were included to gain a deeper understanding of workload-related matters. We included these items to gain further exploratory insights that would complement the MMSS survey questions. While these items were not drawn from validated scales, their content validity was assessed. The survey was reviewed iteratively by members of the research team and by researchers and nurses outside the study team to ensure clarity, contextual relevance, and alignment with the study aims. The survey is included in Supporting 1.

### 2.5. Interview Guide

The interview guide was developed iteratively (Supporting 2) through (1) a literature review to identify the main influencing factors for leaving the nursing profession and (2) discussion with the project team, including members of nursing leadership. This helped to brainstorm topics that were not otherwise identified and validate the content among professional nurses. Furthermore, new discussion topics were added as they emerged during the interviews.

Interviews were conducted by three female health services' researchers trained in qualitative methods (J.S. (PhD), H.W.L. (MSc) or B.W. (PhD)) between 17th Feb and 4th May 2023. Interviews were conducted using a semistructured interview guide and were audio-recorded and transcribed verbatim. Interviews were conducted once, in a quiet place convenient and acceptable to the participant. The interviewers had no pre-existing relationship with the research participants, and anonymity was ensured. We stopped recruitment once no additional insights or information emerged from the interviews. On average, interviews lasted 62 min (range 30–84 min), and participants were compensated $40 for their time.

### 2.6. Data Analyses and Coding

Survey data were analysed using descriptive statistics (means and standard deviations and proportions). The MMSS data are presented in waterfall plots, depicting agreement and disagreement proportions. Likert scale responses were grouped into three categories: satisfied (strongly or partially), neutral and dissatisfied (strongly or partially). A mean MMSS score (0–6) was calculated for each factor to identify the highest and lowest rated job satisfaction items.

Qualitative interview data were analysed using Braun and Clarke's thematic analysis approach [23]. Transcripts were coded line-by-line to reflect the meaning of the sentences and were then consolidated to create subthemes and themes. Coders (J.S., H.W.L. and B.W.) read the transcripts and listened to the audio recordings to familiarise themselves with the data. An initial code book was developed and was continuously reviewed and updated as each transcript was coded. The approach enabled the team to optimise both the consistency of coding and consider reflexivity. Once coding was complete, the team discussed and agreed on the themes. Data were then categorised into micro-, meso- and macrofactors by one researcher (J.S.) and was subsequently verified by two other researchers (B.W. and H.W.L.). Disagreements in coding were discussed between team members until agreement was reached.

To visualise the interactions between the micro-, meso- and macrofactors, a network analysis was conducted using Gephi v10.1. Factors and their interactions were derived from the data and agreed amongst the study team through discussion. Each factor is represented by a single node. The size of the node is determined by the number of connections to other factors; larger nodes representing factors with more connections. The network analysis helped to understand the relationships between the micro-, meso- and macrofactors and the frequency of connections for each factor.

### 2.7. Data Triangulation

To enrich the depth and breadth of our findings, we triangulated survey and interview data [24]. Survey data provided quantitative insights into the prevalence and patterns of factors influencing nurses' decisions to leave the profession, offering a broad understanding of trends within the nursing workforce. Interviews allowed for a qualitative exploration of individual experiences and perspectives. By deep-diving into certain topics, we were able to gain a nuanced understanding of survey data uncovering the underlying reasons behind certain responses. Furthermore, we used triangulation to increase the credibility and validity of our research findings [25].

### 2.8. Analytical Framework

We adopted a system approach to understand motivations for leaving. System thinking describes the relationship between an individual's behaviour, interactions and relationships (microlevel), the organisational environment, including policies and regulations (mesolevel) and the social, economic, political and cultural norms within which an individual and organisation resides (macrolevel) [14]. By using system thinking, we aim to gain a more complete understanding of what influences nursing turnover.

## 3. Results

We received 676 survey responses and interviewed a total of 35 participants (Table 1). For the interviews, we recruited 21 participants through the survey and 14 participants through word-of-mouth. After cleaning the 676 survey responses, we were left with 479 responses in the analyses. Survey data were dropped when participants declined to participate after reading the study outline or had incomplete survey responses. Fifty-three percent of the participants reported that they were thinking about changing their organisation, while 36% were considering leaving the nursing profession. The top five reasons for intending to leave were related to exhaustion (74%), inadequate staffing levels (72%), feeling undervalued (66%), low pay (61%) and excessive pressure (58%).

**Figure 1:**
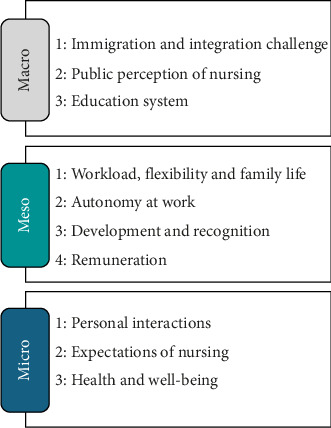
A summary of the key micro-, meso- and macrofactors influencing the decision to leave the nursing profession.

A summary of the key themes identified in the survey and interview data at the micro-, meso- and macrolevels is presented in Figure 1. The subsequent results are presented in four sections: (1) micro-, (2) meso-, (3) macrofactors and (4) micro–meso–macro relationships.  Supporting 3 contains the full McCloskey/Mueller job satisfaction scale scores, and Supporting 4 contains additional supporting quotes from the interview data.

### 3.1. Microfactors

#### 3.1.1. Theme 1: Personal Interactions

Negative nursing cultures were among the most frequently discussed factors, which contributed to a negative work environment and motivated those to leave an organisation.

ID04 “The biggest factor, the reason why they will leave organisations is their manager.” [sic]

Survey data revealed more than 50% were satisfied with nursing peers and the physicians they worked with, reflecting strong professional relationships. However, around a third were dissatisfied with their immediate supervisor and the amount of encouragement and positive feedback they received (Supporting 4).

Negative nursing cultures manifested in various ways, creating a challenging work environment. For example, (as with the survey data) interviewees struggled with unapproachable or unsupportive managers or peers and even described examples of blame culture. Others felt left without confidants and avenues to enact positive change or described situations where they were scrutinised or even penalised when requesting time off.

ID26 “Why you never go for a break? Yeah, it means your time management is not good enough.” [sic]

ID06 “We can't even share with our RO [Reporting Officer], because there'll be issues, you won't have peace in working” [sic]

ID03 “When you are asking for family care leave, your kids are not well and so on, they were asking you “are you sure? Why always not well? These kind of words making you feel frustrated.” [sic]

Generational differences also shaped nursing cultures. Interviewees described younger workers as seeking greater transparency and understanding in their roles, often being more vocal about their needs. In contrast, older nurses were often described as employing traditional management styles, characterised by straightforward instructions and limited encouragement or explanation. These differences created workplace tensions.

ID21 “[Older generation] I think they won't really understand the younger generation what they are thinking, that's what I see. Because when I tell my manager about something they will say no it must be like this.” [sic]

ID01 “I think a lot of the senior nurses will agree with me that, the new nurses now, they have very different expectations.” [sic]

Another example influencing nursing cultures is personal biases, manifesting as gender discrimination, ethnic group disparities and workplace cliques.

ID22 “So I think there's some kind of uh bias towards female nurses.” [sic]

ID20 “We do feel there's some favouritism towards the nationality.” [sic]

Finally, foreign nurses frequently encountered communication challenges and the stress of learning a new language, complicating their transition and integration into the country and workplace.

ID09 “At first I also had hard time. Yeah. Then slowly, slowly then just learn…”

ID12 “You know I can't communicate, you know, with patient in Cantonese or Malay or Hokkien.” [sic]

#### 3.1.2. Theme 2: Expectations of Nursing

For some individuals, the reality of the nursing profession did not meet their expectations, leading to disillusionment and a desire to leave the field. Some noted that unrealistic portrayals of nursing are unhelpful, while others observed that students sometimes use nursing as a stepping stone or consider it a last-resort choice.

ID18 “So everyone has this idea, you see all the advertisements and all that and it's really not like that.” [sic]

ID01 “They just want to get the diploma paper then jump out and move on to other career.” [sic]

#### 3.1.3. Theme 3: Health and Well-Being

Many nurses struggled with poor health and well-being due to work fatigue and the mental toll of their profession. This strain led to high rates of medical leave, exacerbated by pressure to work while unwell. Survey data revealed 62% felt under too much pressure while at work and 57% felt pressure to work when unwell.

ID I9 “I think it took a toll on my friendships and my personal life. I had very bad insomnia because there were like three different shifts to handle…I just felt it taking a toll on my health.” [sic]

### 3.2. Mesofactors

#### 3.2.1. Theme 1: Workload, Flexibility and Family Life

**Figure 2:**
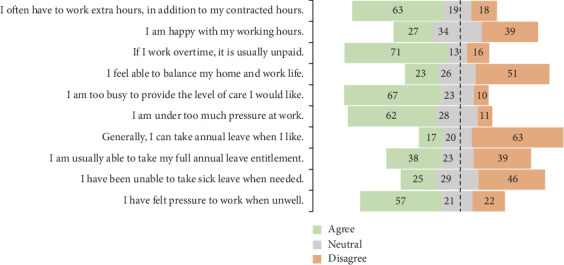
Waterfall plot of opinions around remuneration, workload and the work environment (proportion (%) agree, neutral and disagree).

Interviewees often described overwhelming workloads and excessive administrative tasks. Over 60% agreed that working extra hours was common and 71% agreed that overtime is usually unpaid (Figure 2).

ID22 “All the documentation work is sucking a lot of time…the responsibility of doing document is like, I'm not a nurse I'm a ward clerk.” [sic]

Heavy workloads led to poor work-life balance. About half agreed/strongly agreed they could not balance home and work life, and over 60% reported they could not take annual leave when desired (Figure 2).

ID15 “I got money but no life…” [sic]

Nursing staff desired flexible working, but many found it unobtainable amidst staff shortages. Survey responses (Supporting 3) reflected poor availability of flexible work options, with opportunity for part-time work (mean score 2.65/5) and flexible work schedules (mean score 2.58/5), amongst the lowest rated job satisfaction items.

ID30 “It'll be difficult, we always don't have enough nurses covering the full-time work, where do we get the part-time nurses.” [sic]

#### 3.2.2. Theme 2: Autonomy at Work

Control over work was one of the lowest rated survey items (mean score 2.69/5), with 43% reportedly dissatisfied with control in their work setting. Interviewees frequently found themselves lacking independence in decision-making. Many viewed the entrenched traditional practices, hierarchical mindsets and the presence of older generations upholding these norms as significant barriers to change.

ID17 “In Singapore it's like doctor is above the rest. So it's like, oh if you're a doctor I need to please you.” [sic]

#### 3.2.3. Theme 3: Development and Recognition

**Figure 3:**
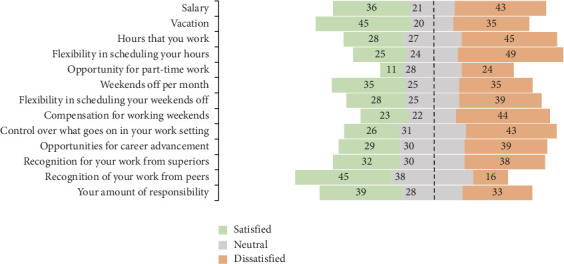
Waterfall plot of selected mesolevel factors captured in the McCloskey/Mueller Job Satisfaction Scale (proportion (%) rated satisfied, neutral and dissatisfied).

Barriers to development and advancement were a key driver for leaving, with 39% reporting dissatisfaction with opportunity for advancement (Figure 3). Despite recognition for work being rated highly (among the top five rated items in the job satisfaction scale), developmental barriers and a lack of recognition were reflected in the interviews.

ID15 “I'm not promoted then question myself, is it because of me? I decided to resign because I wasn't promoted.” [sic]

ID32 “They actually put on more responsibility for us, but they do not want to kind of fairly recognise us for it.” [sic]

#### 3.2.4. Theme 4: Remuneration

More than 40% of survey participants were unhappy with their salary overall and weekend compensation (44%) (Figure 3). Interviews revealed mixed feelings towards remuneration, with dissatisfaction stemming from unpaid overtime and poor compensation for unsociable working times such as night shifts and weekend work. In contrast, vacation benefits were rated more highly (45% satisfied).

ID13 “The risk that we take are quite high actually, so I think remuneration needs to be high.” [sic]

### 3.3. Macrofactors

#### 3.3.1. Theme 1: Immigration and Integration Challenges

Nurses from overseas faced a multitude of issues both personally and professionally including issues sourcing accommodation and the cultural shock of living and working abroad. These challenges placed a significant burden on foreign nursing staff.

ID06 “Having transitioned into a community, it is a culture shock…but yeah the hospital expects us to you know just get it our way and you know settle ourselves.” [sic]

Furthermore, strict immigration policies prevent foreign nurses from bringing their families or obtaining permanent residency status, which have implications for their long-term stay.

ID02 “A lot of them I'm hearing is actually because of family [leaving Singapore] they are separated from their family and like being in, for example New Zealand, they can easily bring their family over.” [sic]

#### 3.3.2. Theme 2: Public Perception of Nursing

Nurses frequently recounted feelings on the poor public image of nursing and unreasonable patient expectations. Nurses described an ecosystem, starting at school and continuing into practice, where nursing is deemed undesirable and a ‘nonprofessional' role.

ID27 “But there's a trajectory that Singapore puts its students on. That sort of funnels them, like to be someone you got to do ABC, and nursing is not ABC.” [sic]

ID32 “Patients do think that for all the allied health professions they are smarter I'd say and they're more seen like a professional help than as a aid [comparison to nurses].” [sic]

ID29 “My parents are questioning my choice as well, how come you learn you want to go this pathway [nursing].” [sic]

The public perception of nursing often subjected nurses to derogatory remarks, abuse and an increased burden of non-nursing tasks, increasing their workload.

ID02 “It's a cultural expectation. So the caregivers, or the next of kin or family member expect very like-we are the maid.” [sic]

#### 3.3.3. Theme 3: Education System

Nurses described their training and implications for the next generation of nurses. Degree holders were seen as having different expectations of their role and less bedside experience compared to diploma nurses. In addition, degree holders often expected faster promotion into leadership roles, leading to tensions among staff.

ID18 “So they are also not so willing, I won't say all, but I would say 70%–80% of the degree holder nurses are more academic and I think they are more interested to do research than really nursing work.” [sic]

### 3.4. Micro–Meso–Macro Relationships

**Figure 4:**
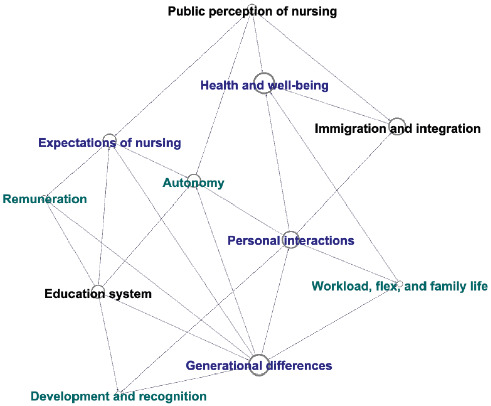
Micro–meso–macro (blue, green and black, respectively) relationships (larger circles indicate more connections between factors).

Figure 4 highlights the micro-, meso- and macrolevel relationships that have been identified. Personal interactions (micro) and generational differences (micro) were found to be pivotal factors, influencing other domains such as health and well-being (micro), workload, flex and family life (meso), development and recognition (meso), autonomy (meso) and immigration and integration (macro). Not all factors interacted across the micro-, meso- and macrolevels. For instance, connections with personal interactions were limited to micro- and mesolevel factors, while generational differences, the education system and public perception of nursing spanned the micro-, meso- and macrolevels.

## 4. Discussion

We conducted a mixed-method study to understand factors influencing nurses' decision to leave the profession. Results showed a complex interplay of micro-, meso-, and macrofactors shaping the nursing experience. Survey data revealed dissatisfaction with work-life balance, poor autonomy and remuneration. Over half of the participants were considering leaving their organisation, and a third were contemplating exiting the profession due to exhaustion, understaffing, feeling undervalued, low pay and excessive pressure. Qualitative interviews revealed negative personal interactions, generational conflicts, unmet expectations of nursing (microlevel), limited autonomy, administrative burdens, poor work-life balance (mesolevel), integration challenges, prohibitive immigration policies for foreign nurses, negative public perceptions and the impact of education on nursing expectations (macrolevel). By taking a systems' approach, we have gained a more holistic understanding of what impacts nurses' decision to leave.

Several factors that we have identified as influencing decisions to leave, such as peer relationships, work-life balance, career progression and administrative burden, are common globally [4–6, 26–28], emphasising their importance in workforce retention. Our qualitative findings also highlighted generational conflict as a source of workplace tension, particularly around differing work values, communication styles and expectations. This aligns with the literature suggesting that generational diversity in the nursing workforce—while potentially enriching—can contribute to interpersonal conflict and dissatisfaction when not well managed [29, 30]. Strategies such as intergenerational mentorship and communication training have been proposed to bridge these divisions [29, 30].

Other insights were more contextual such as how multiculturism affects the nursing workforce, including its influence on retention. We found language and cultural barriers, treatment bias, segregation from family and barriers to career progression due to residency status undermined team cohesion, created inequalities and dissatisfaction among foreign-trained nurses. Stahl and Maznevski suggest that while diverse teams' may experience challenges, such as lower social integration and higher task conflict, multicultural teams gain from enhanced creativity and problem-solving skills [31]. Thus, to leverage these advantages, building improving cultural intelligence within teams is critical to improve team cohesion and performance [32]. Other suggested strategies include offering permanent residency, providing equal opportunities for career advancement, tailoring induction programmes and enabling residency for family members, all of which can help integrate foreign nurses into the workforce [13].

Another important consideration in workforce retention is autonomy in practice. Survey and interview responses indicate poor professional autonomy and a desire for more independence in their practice. Nurses reflected in the interviews that traditional mindsets and the hierarchical nature of many Asian cultures were barriers to autonomy, in contrast to Western cultures which emphasise individual freedom [33]. Greater perceived autonomy is associated with improved job satisfaction [34, 35], so further improvement in this domain is essential in positively influencing retention.

Efforts to shift towards greater and team-based care include the Advanced Practice Nursing role introduced in 2003 [36]. In Singapore, Advanced Practice Nurses are equipped with the capabilities to independently perform a broad range of comprehensive healthcare services, such as recommending and ordering diagnostic tests and prescribing medications. Their advanced education and clinical training allow them to provide specialised care and collaborate with doctors to provide holistic patient care [37]. Another example is the Integrated General Hospital model at Alexandra Hospital, which was established in 2018, fostering team-based rather than traditional hierarchical-orientated care. In addition, this care model empowers nurses by emphasising nurse-led care for low-acuity patients while optimising physician contact for higher-acuity patients [38].

Our study identified several factors influencing workforce retention, including negative workplace cultures, poor public perception, overwhelming and inflexible workloads and limited autonomy, as reflected in our survey and interview data. We also revealed how micro-, meso- and macrolevel factors interact to shape work experience. For example, macroeducational factors influence microlevel role expectations and macrolevel public perceptions of nursing, which in turn affect the microlevel health and well-being of nurses. By adopting a system perspective, we can develop holistic interventions that address these multifaceted challenges.

Our findings suggest several implications for practice. At the organisational level, interventions should target modifiable mesolevel drivers such as work-life balance, administrative burden and autonomy. Strategies such as flexible scheduling, improved staffing models and streamlining documentation through digital tools may help alleviate workload pressures and enhance job satisfaction. To address negative workplace cultures and intergenerational tensions, healthcare institutions should invest in leadership development, peer support programmes and training in cultural intelligence. At the policy level, rethinking immigration pathways and professional recognition for foreign-trained nurses could enhance retention in multicultural systems, such as Singapore's. Public perception campaigns may also help reframe nursing as a skilled, autonomous and vital profession—a key to tackling the global image problem that contributes to attrition. Future research should explore the effectiveness of such interventions longitudinally and investigate the role of culturally competent leadership in diverse teams. Additionally, longitudinal studies may help clarify how professional expectations evolve over time and how macropolicy reforms translate into day-to-day experiences on the ground.

### 4.1. Strengths and Limitations

This mixed-method study has many strengths and limitations that should be acknowledged. First, we attempted to snowball our survey to improve the representativeness of the responses. We were able to analyse 479 responses, representing a fraction of Singapore's nursing workforce. Furthermore, using an electronic survey format may have biased the sample to those who are more technology literate. With regard to the interviews, while we continued to recruit participants until no new themes emerged, our sample may not represent users in all nursing settings. Finally, this was a cross-sectional study; thus, we were unable to explore the longitudinal changes in topics such as job satisfaction.

## 5. Conclusions

Our study underscores the importance of adopting a multifaceted system approach to understand and address nurse retention issues comprehensively. We identified micro-, meso- and macrolevel factors and the interplay between them. Recognising and addressing these complexities will be essential in developing future interventions to improve nurse retention.

## Figures and Tables

**Table 1:** Characteristics of survey and interview participants.

Demographics	Survey	Interviewees	
	*n* = 479	Current nurse *n* = 20	Ex nurse *n* = 15
Age in years, mean (SD)	35.5 (SD 8.9)	36.5 (8.8)	39.1 (8.8)
Gender, female *n* (%) (*n* = 439 survey responses)	405 (92)	14 (70)	13 (87)
Race *n* (%), (*n* = 429 survey responses)			
Chinese	250 (58)	14 (70)	10 (66)
Indian	41 (10)	3 (15)	1 (7)
Malay	61 (14)	1 (5)	3 (20)
Others	77 (18)	2 (10)	1 (7)
Marital status *n* (%)			
Single	224 (47)	10 (50)	3 (20)
Married	255 (53)	9 (45)	12 (80)
Not reported	0 (0)	1 (5)	0 (0)
Residency status			
National/permanent resident	—	14 (70)	15 (100)
Foreigner	—	6 (30)	0 (0)
Full-time employment *n* (%)	472 (99)	20 (100)	20 (100)
Job title *n* (%) (*n* = 476 survey responses)			
Junior (e.g., EN, principal EN, senior EN)	48 (10)	1 (5)	1 (7)
Mid-level (e.g., staff nurse, senior staff nurse, asst. nurse clinician)	390 (82)	13 (65)	14 (93)
Senior (e.g., nurse clinician, nurse manager, advanced practice nurse, director)	38 (8)	6 (30)	0 (0)
Years of experience *n* (%)			
0 to 5	114 (24)	4 (20)	13 (87)
6 to 10	131 (27)	2 (10)	2 (13)
11 or more years to 15	234 (49)	14 (70)	0 (0)

Abbreviations: EN, enrolled nurse; SD, standard deviation.

## Data Availability

The data that support the findings of this study are available from the corresponding author upon reasonable request.

## Nomenclature

EN: Enrolled nurse
COREQ: Consolidated Criteria for Reporting qualitative Research
CROSS: Consensus-Based Checklist for Reporting of Survey Studies
MMSS: The McCloskey/Mueller Satisfaction Scale
NUHS: National University Health System
SD: Standard deviation

## Supporting Information

Supporting Information 1 Supporting 1: Job satisfaction questionnaire.

Supporting Information 2 Supporting 2: In-depth Interview Topic Guide: Current nurses and Ex nurses.

Supporting Information 3 Supporting 3: The McCloskey/Mueller Satisfaction Scale, proportion satisfied to dissatisfied (%).

Supporting Information 4 Supporting 4: Themes and additional accompanying quotations from the interviews.
