# Supplementary material for: Micro-, Meso- and Macrofactor Relationships in Nursing Turnover: Insights From Survey and Interview Data
Source: J Nurs Manag. 2025 Jul 1;2025:5078305. doi: 10.1155/jonm/5078305 (PMC12237555; doi:10.1155/jonm/5078305)
Supplement: Supporting Information 1 — Supporting 1: Job satisfaction questionnaire. [file 5078305.f1.pdf]

Supplement 1: Job satisfaction questionnaire

<u>Job satisfaction</u>

1. **Please rank your agreement with each of the following topics**

<table>
<tr><th>Very satisfied</th><th>Moderately satisfied</th><th>Neither satisfied nor dissatisfied</th><th>Moderately dissatisfied</th><th>Very dissatisfied</th><th>I choose not to respond</th><th>Not applicable</th></tr>
<tr><td colspan="7">

- Salary
- Paid leave (no. of days of annual leave, medical leave, family care leave etc.)
- Benefits package (insurance, medical benefits, retirement)
- Hours that you work
- Flexibility in scheduling your hours
- Opportunity to work on consecutive days
- Opportunity for part-time work
- Weekends off per month
- Flexibility in scheduling your weekends off
- Compensation for working weekends
- Duration of Maternity leave
- Child care facilities within the institution
- Your immediate supervisor
- Your nursing peers
- The physicians you work with
- The method of delivering nursing care in your unit (e.g., functional, team, primary)
- Opportunities for socializing with colleagues at work
- Opportunities for socializing with your colleagues after work
- Opportunities to interact professionally with colleagues from other disciplines
- Opportunities to interact with faculty in nursing schools i.e. NYP/NP/NUS/SIT
- Opportunities to belong to ward-level, department-level and/or institutional-level committees
- Control over what goes on in your work setting
- Opportunities for career advancement
- Recognition for your work from your superiors
- Recognition for your work from peers
- Amount of encouragement and positive feedback
- Opportunities to participate in nursing research
- Opportunities to write and publish
- Your amount of responsibility
- Your control over work conditions
- Your participation in organization-level decision making

</td></tr>
</table>

Workload

2. **Please rate your agreement with the following statements**

Strongly agree	Agree	Neither agree nor disagree	Disagree	Strongly disagree

- I often have to work extra hours, in addition to my contracted hours
- I am happy with my working hours
- If I work overtime, it is usually unpaid
- I feel able to balance my home and work life
- I am too busy to provide the level of care I would like
- I am under too much pressure at work
- Generally, I can take annual leave when I like
- I am usually able to take my full annual leave entitlement
- I have been unable to take sick leave when needed
- I have felt pressure to work when unwell

Less than an hour	1-2 hours	3-6 hours	7-10 hours	Over 10 hours	NA
**3. On average, how many extra PAID hours do you work a week, in addition to your contracted hours?**					
○	○	○	○	○	
**4. On average, how many UNPAID hours do you work a week, in addition to your contracted hours?**					
○	○	○	○	○	

Benefits

5. **Please rate your agreement with the following statements**

Strongly agree	Agree	Neither agree nor disagree	Disagree	Strongly disagree

- Given my role and responsibilities, I feel my current pay band/grade is appropriate
- I feel there are better nursing opportunities abroad
- I feel there are limited nursing opportunities locally

6. **I am dissatisfied with my pay band/grade because (check all that apply):**

Failed to keep up with in the increased cost of living
Dissatisfaction with the organisation's pay structures
Perceived failure to reward nursing staff fully for their effort and contribution
I am not paid for overtime
I am not adequately compensated for weekend or night work
Other (Please specify: ________________________________)

Intention to leave

7. **For each of the statements below, how often, if at all, do these statements apply to you?**

**Strongly agree**	**Agree**	**Neither agree nor disagree**	**Disagree**	**Strongly disagree**
• I am thinking about changing organization but will continue nursing.<br>• I am thinking about leaving the nursing profession.				

8. **Sometimes I feel like leaving the nursing profession because (check all that apply):**

- Feeling undervalued
- Too much pressure
- Feeling exhausted
- Staffing levels are too low
- Levels of pay are too low
- Can’t give the level of care or standard I would like
- Not enough managerial support
- My own stress levels or poor health
- Too much paperwork/ bureaucracy
- Looking for a new challenge
- Retirement
- Unable to progress my career
- Not applicable

9. **Demographics**

**Gender:**	o	Female	o	Male	o	Prefer not to disclose	o	Others		
**Age:**										
**Race:**	o	Chinese	o	Indian	o	Malay	o	Others (please specify):		
**Marital status:**	o	Single	o	Married	o	Divorced				
**Highest educational level:**	o O-level<br>o N-level<br>o A-level<br>o Diploma<br>o Advanced diploma<br>o Bachelor degree<br>o Post-graduate degree									
**Employment status:**	o	Full-time	o	Part-time	o	Student				
**Are you a regular caregiver for any children?**	o	Yes	o	No						
**Are you a regular caregiver for family members (not children), friends or neighbors?**	o	Yes	o	No						
**In general, how would you rate your overall health?**	o	Excellent	o	Very good	o	Good	o	Fair	o	Poor
**Job title:**	o Enrolled nurse<br>o Principal enrolled nurse				o Staff nurse<br>o Senior staff nurse		o Asst. Nurse Clinician<br>o Nurse Clinician<br>o Senior Nurse Clinician<br>o Nurse Educator<br>o Nurse Manager<br>o Senior Nurse Manager<br>o Advanced Practice Nurse<br>o Other (please specify)			

**Years of experience in nursing:**		
**I currently work in:**	o Public hospital<br>o Community hospital	o Private hospital<br>o General practitioners/ polyclinic<br>o Others: please specify _________________________
**Clinical area of employment:**	o Inpatient wards<br>o Outpatient clinics	o Urgent care clinic/ Emergency department<br>o Intensive care/high dependency units<br>o Others: please specify _________________________

10. **Any other points you wish to raise? ____**
11. **Would you or a colleague like to take part in an in-depth interview on your experiences of nursing and why you might leave the profession? If yes, please click on this link to confidentially share your contact information**
