# Supplementary material for: Micro-, Meso- and Macrofactor Relationships in Nursing Turnover: Insights From Survey and Interview Data
Source: J Nurs Manag. 2025 Jul 1;2025:5078305. doi: 10.1155/jonm/5078305 (PMC12237555; doi:10.1155/jonm/5078305)
Supplement: Supporting Information 2 — Supporting 2: In-depth Interview Topic Guide: Current nurses and Ex nurses. [file 5078305.f2.pdf]

Supplement 2

In-depth Interview Topic Guide: Current Nurses

Nursing experience

1. Why did you choose to become a nurse?
2. Have your expectations of nursing been different from reality?
3. What have you liked and disliked about nursing so far?
4. How do you see your career progressing?
5. Have you ever thought about moving to a different nursing role?
6. Have you ever thought about leaving the nursing profession?
7. Is there anything that could improve your position?
8. What doesn't need improving and makes you want to continue as a nurse?

Impact of Covid-19

9. Were you working as a nurse during COVID-19? How was that experience?
10. How did Covid-19 impact your role?

Debrief

11. Do you have any other comments?

In-depth Interview Topic Guide: Ex Nurses

Past experiences of being a nurse

1. Why did you choose to become a nurse?
2. Were your expectations of nursing different from reality?
3. What have you liked and disliked about nursing?

Reasons for leaving

4. How long had you considered leaving nursing when you were practising?
5. What made you decide to leave? Was there an event that led to this decision?
6. Was anything done to change your mind about leaving?

Impact of Covid-19

7. Were you working as a nurse during COVID-19? How was that experience?
8. Did it impact your decision to leave the profession?

Probing solutions

9. Would you consider returning to nursing in the future?
10. What would need to change for you to consider nursing as a career again?
11. What are your thoughts on the future of the nursing profession?

Debrief

12. Are there any other points you would like to raise or discuss?
