# Supplementary material for: Micro-, Meso- and Macrofactor Relationships in Nursing Turnover: Insights From Survey and Interview Data
Source: J Nurs Manag. 2025 Jul 1;2025:5078305. doi: 10.1155/jonm/5078305 (PMC12237555; doi:10.1155/jonm/5078305)
Supplement: Supporting Information 3 — Supporting 3: The McCloskey/Mueller Satisfaction Scale, proportion satisfied to dissatisfied (%). [file 5078305.f3.pdf]

Supplement 3: The McCloskey/Mueller Satisfaction Scale, proportion satisfied to dissatisfied (%)

Question	1	2	3	4	5	6	7	8	9	10	11	12	13	14	15	16	17	18	19	20
**very satisfied**	5.2	7.5	3.5	5.2	3.8	3.8	3.1	9.8	7.1	2.3	2.5	1.5	13.8	13.4	5.4	6.1	7.3	5.4	2.7	3.1
**moderately satisfied**	30.9	37.2	25.5	22.5	21.5	20.5	7.5	24.6	20.7	7.9	10.2	9.0	28.6	44.9	47.0	38.6	41.1	30.7	32.2	21.5
**neutral**	21.1	19.8	25.9	27.3	23.8	41.8	27.6	25.1	24.8	10.6	18.4	25.7	29.2	28.0	30.9	31.7	33.0	39.9	39.2	37.2
**moderately dissatisfied**	30.1	23.4	30.3	26.9	28.4	19.0	12.5	19.8	22.8	8.8	12.9	6.3	15.7	10.0	12.1	17.3	11.1	12.1	16.1	9.4
**very dissatisfied**	12.7	12.1	14.6	17.7	20.9	9.6	11.7	14.6	15.9	9.2	12.7	9.2	12.5	3.8	4.4	6.3	6.7	8.4	6.7	5.4
**NA**	0.0	0.0	0.2	0.2	1.7	5.4	37.6	6.1	8.8	61.2	43.2	48.4	0.2	0.0	0.2	0.0	0.8	3.5	3.1	23.4

Question	21	22	23	24	25	26	27	28	29	30	31
**very satisfied**	2.1	1.5	5.2	5.0	6.7	5.2	3.1	2.3	4.8	1.7	2.3
**moderately satisfied**	31.3	24.2	23.6	26.5	38.4	29.2	13.6	8.8	34.0	25.5	18.2
**neutral**	43.4	31.3	30.1	30.1	38.2	34.0	39.9	39.2	28.0	31.3	40.5
**moderately dissatisfied**	10.6	27.3	22.5	20.0	10.9	17.7	10.0	10.2	20.9	24.0	16.7
**very dissatisfied**	6.7	15.2	16.1	17.5	5.2	13.2	10.4	9.8	11.9	16.9	14.0
**NA**	5.8	0.4	2.5	0.8	0.6	0.6	23.0	29.6	0.4	0.6	8.4
