# Supplementary material for: Micro-, Meso- and Macrofactor Relationships in Nursing Turnover: Insights From Survey and Interview Data
Source: J Nurs Manag. 2025 Jul 1;2025:5078305. doi: 10.1155/jonm/5078305 (PMC12237555; doi:10.1155/jonm/5078305)
Supplement: Supporting Information 4 — Supporting 4: Themes and additional accompanying quotations from the interviews. [file 5078305.f4.pdf]

Supplement 4: Themes and additional accompanying quotations from the interviews

Factors	Themes	Quotations
Micro	1. Personal interactions	*"I also encountered this kind of colleagues whereby they are not receptive, they don't want to help you. Even sometimes to a point where they sabotage you or your work and everything [sic]"* (ID31)<br><br>*"when I actually shared with them [management] all these difficulties and concerns that I have, they actually said that I should understand my managers, because they are handling a lot of stuff. But I don't think that is very good excuse, I think they should also understand where we are coming from [sic]"* (ID32)<br><br>*"in many aspects TPM [annual performance appraisal] here mistaken as a axe to grind. They talk about your mistakes, they talk something happened many years ago, talk about medical leave. [sic]"* (ID01)
	2. The effects of age, gender, and ethnicity	"*and in terms of favouritism, yeah, I think it's very prominent. I could see it towards like my male colleagues to my boss, my RO [reporting officer], towards my male colleagues versus like my female colleagues [sic]"* (ID13)<br><br>*"so why I want to transfer to XXX, if you are not in that [racial] clique, you will be stagnant [sic]"* (ID33)<br><br>*"Some of the nurses are like those, how to say, old school. If you do something wrong, then they will scold you…Juniors nowadays, they will talk, they are very direct…so you have to adapt with different generation and the way you interact with them has to be different [sic]"* (ID16)
	3. Expectations of nursing	*"I did hear a lot of stories before I graduated. So like, it prepared me a bit, but I didn't expect the workload to be so much until I don't have time to go for a break or go toilet, it was over whelming [sic]"* (ID19)<br><br>*"but I guess most of these people who joined nursing, this was their last resort [sic]" (ID02)*
	4. Health and well-being	*"that was why I left after eight, nine years, it was too tiring [sic]"* (ID18)<br><br>*"There's high MC rate in the ward. People are being exhausted [sic]"* (ID22)<br>*"I come back, not fully recovered, and like some people are working still like who didn't take MC at all are also sick. Like the patients look way better than us [sic]"* (ID27)

Meso	1. Workload, flexibility, and family life	*"our overtime wasn't optional it was mandatory because they already scheduled us in the roster [sic]"* (ID31)<br><br>*"like keep on like on task after another task, you cannot finish, you don't even have time to go for your break [sic]"* (ID10)<br><br>*"if flexible hours is a choice, then ok, who wouldn't want that? But I don't think that's possible in nursing [sic]"* (ID34)
	2. Autonomy at work	*"the Singapore system has been like that for many years. Even though we are trying to make the nurses do more….the older generation is still around. [sic]* (ID18)<br><br>*"[nurses with more autonomy] I don't know because is really cultural difference, I think we are too set up with old culture. [sic]"*
	3. Development and recognition	*"I'm not promoted then question myself, is it because of me? I decided to resign because I wasn't promoted [sic]"* (ID15)<br><br>*"so I think all these are important are really driving forces that nurses are not staying in Singapore because of long working hours, poor working environment, poor reputation of nurses, not enough recognition [sic]"* (ID13)
	4. Renumeration	*"Like in Australia you get penalty rates for working overtime, penalty rates for public holidays, weekends…..we do got that here but financially speaking that's not worth my time [sic]"* (ID27)
Macro	1. Immigration and integration challenges	*"so actually sometimes the landlords don't like shift workers [sic]"* (ID21)<br><br>*"it really needs a lot of time to learn and to adapt. Its not like you have a registered nursing practice you can immediately practice. It needs time to adapt [sic]"* (ID10)<br><br>*"plus the culture, also language. Oh I think that time is very stressful [sic]"* (ID12)<br><br>*"They've applied multiple times for like PR [permanent residency], you know….and they still haven't gotten, they're got rejected so many times, and to me that's ridiculous [sic]"* (ID27)
	2. Public perception of nursing	*"it's not merely the workload..i guess the mindset and the perception we have of nurses in general, where they still see*

	nurses as people that have to do every single little thing for the patients [sic]" (ID24)
3. Educating the next generation	*"It's much more theory and lectures to be frank. So when they graduate their mindset is different as well [Bachelor-prepared versus Diploma-prepared] [sic]"* (ID33)
